# Some Aspects of Eye Disease in the Transkei

**Published:** 1980

**Authors:** J. D. Huggan

**Affiliations:** Senior Registrar, Bristol Eye Hospital


### Some Aspects of Eye Disease in the Transkei

(A short summary of a paper of the same title based on ophthalmic work done in the Transkei, Southern Africa, from 6th November to 13th December 1978)

**J. D. Huggan, F.R.C.S.E.**
Senior Registrar, Bristol Eye Hospital

The purpose of this project was to be a study of acute eye conditions in the Bantu Negro of Southern Africa, and to draw attention to ways in which such conditions might vary from Caucasians. Such comparisons were found to be difficult, the main reason being that there was often a great delay before treatment was sought, so that direct comparison could seldom be applied. The vast bulk of ocular pathology came from injuries, cataract and disease of the cornea, these three categories together comprising 70% of the 140 patients seen and treated in Umtata, where the only permanently established eye clinic in the country, staffed by one Ophthalmologist, exists. With the exception of cataract, the number of patients attending with any one condition was fairly small and the project was expanded to analyse the entire gamut of conditions presenting.

Trauma was generally a serious problem in patients presenting with it, and many eyes were already rendered useless on presentation. Infection, although a serious problem, did not cause as much visual loss as iritis and binding down of the pupil to a cataractous lens.

Corneal disease presented in a wide variety of ways and it seems clear that infection and a variety of poorly understood racial and, possibly, environmental factors are responsible for the difference in spectrum between corneal disease here and in, for example, the UK.

Cataract is said to occur with equal incidence in white and black populations in Southern Africa, but it is almost certain that there is a greater prevalence of the condition in the Bantu simply because of less treatment, i.e. cataract extraction being available. With one exception the patients seen and operated on for cataract had dense mature blinding cataract in both eyes. Two patients had cataract caused by lightning.

There was a variety of other conditions ranging from conjunctivitis of various types through severe iritis and assorted retinal disease to advanced glaucoma and grossly advanced orbital ocular and lid tumours. Many of these required referral to the department in Durban for further investigation and /or treatment not available in Umtata.

Relatively little is written about exogenous iritis in the Negro, but in those dealing with Negro patients it is well known that they appear to suffer a more severe reactive iritis to stimuli such as trauma and surgery. This is often attributed, for example, to the increased amount of pigment in the eye but why this should be is not clear. It has been shown that Negroes have a reduced level of adrenocorticoid activity and it has been suggested therefore that this is why Negroes in general respond to trauma or surgery with a more exuberant tissue reaction involving a greater degree of granulomatous inflammation and gamma-globulin synthesis. It is highly interesting to speculate that the blood-aqueous barrier in the Negro is more easily broken down for this reason.

The assistance of the BRI Hospital Medical Executive Committee in facilitating this project was greatly appreciated as this trip turned out to be a marvellous professional and educational experience.

